# Can the Substitution of Milk with Plant-Based Drinks Affect Health-Related Markers? A Systematic Review of Human Intervention Studies in Adults

**DOI:** 10.3390/nu15112603

**Published:** 2023-06-01

**Authors:** Paola Biscotti, Cristian Del Bo’, Catarina Carvalho, Duarte Torres, Emmanuelle Reboul, Beatrice Pellegrini, Valentina Vinelli, Angela Polito, Laura Censi, Marisa Porrini, Daniela Martini, Patrizia Riso

**Affiliations:** 1 Department of Food, Environmental and Nutritional Sciences (DeFENS), Università degli Studi di Milano, 20133 Milan, Italy; 2 EPIUnit—Instituto de Saúde Pública, Universidade do Porto, 4050-600 Porto, Portugal; 3 Laboratório Para a Investigação Integrativa e Translacional em Saúde Populacional (ITR), 4050-600 Porto, Portugal; 4 Faculdade de Ciências da Nutrição e Alimentação, Universidade do Porto, 4150-180 Porto, Portugal; 5 Aix-Marseille Université, INRAE, INSERM, C2VN, 13885 Marseille, France; 6 Council for Agricultural Research and Economics-Research Centre for Food and Nutrition, 00178 Rome, Italy

**Keywords:** sustainability, plant-based beverages, health, intervention studies

## Abstract

The consumption of plant-based drinks (PBDs) in substitution for cow’s milk (CM) is increasing due to concerns for human and planet health and animal welfare. The present review aims to analyze the main findings from intervention trials investigating the effect of PBDs in comparison with CM on markers of human health. Suitable articles published up to July 2022 were sourced from PubMed and Scopus databases. A total of 29 papers were collected, with 27 focusing on soy drinks (1 of which also evaluated the effects of an almond drink), while only 2 focused on rice drinks. Among studies focused on soy drinks, the most investigated factors were anthropometric parameters (*n* = 13), the lipid profile (*n* = 8), markers of inflammation and/or oxidative stress (*n* = 7), glucose and insulin responses (*n* = 6) and blood pressure (*n* = 4). Despite some evidence of a beneficial effect of PBDs, especially for the lipid profile, it was not possible to draw any overall conclusions due to some conflicting results. As well as the low number of studies, a wide heterogeneity was found in terms of the characteristics of subjects, duration and markers, which reduces the strength of the available results. In conclusion, further studies are needed to better elucidate the effects of substituting CM with PBDs, especially in the long term.

## 1. Introduction

By virtue of its nutritional value, particularly the contents of high-biological-value proteins and calcium (Ca), the consumption of cow’s milk (CM) is highly recommended in most dietary guidelines [1]. Nevertheless, Western countries are facing a remarkable reduction in its consumption; for example, from 2005–2006 to 2017, the number of Italian milk consumers decreased from 75% to 61%, and in France, the number of consumers decreased from 70% to 57% from 2007 to 2014, while in the Netherlands, it declined from 65% to 56% from 2003 to 2012 [2]. Several reasons may explain the reduction in or even the exclusion of milk and dairy products from the diet, including real or perceived allergies and lactose intolerance [3] and sometimes health concerns. Beyond these reasons, environmental concerns and ethical considerations regarding animal welfare are also influencing this reduction [4]. Because of the growing adoption of milk-free diets, consumer demand for plant-based drinks (PBDs) has been increasing worldwide over the years. The global market for PBDs is forecast to reach USD 40.6 billion by 2026, with an annual growth rate of 10.3% [5].

To date, there is no agreed definition or classification of these PBDs. However, it is possible to consider a classification based on the sources used in their production: cereals (oat, rice, corn, spelt), legumes (soy, peanut, lupin, cowpea), nuts (almond, coconut, hazelnut, pistachio, walnut), seeds (sesame, flax, hemp, sunflower) and pseudo-cereals (quinoa, teff, amaranth) [6]. The production processes of PBDs include several steps and the addition of ingredients such as salt, sugar and flavorings, which are often used to fulfill the consumer demand for a product that resembles CM in terms of color, texture and flavor [7,8,9]. Due to the presence of substances such as hydrolyzed proteins, emulsifiers, emulsifying salts, added salt and fat, 90% of PBDs from the USDA Branded Food Products Database are considered ultra-processed foods [10]. Fortification is mostly performed with Ca and vitamin D, but sometimes ingredients such as zinc and other vitamins (e.g., A, E and B_12_) are also added. Among the added ingredients, PBDs can also be supplemented with proteins (usually isolated or extracted from sources such as peas or soy) [7,8,9,10,11]. Therefore, despite being included in the same category, the nutrient composition of PBDs can differ considerably according to their source and product formulation.

In general, not all PBDs are comparable or equivalent to CM in terms of nutritional composition [12,13,14,15,16,17,18]. In detail, relevant differences in terms of macronutrient and micronutrient contents between most PBDs and CM have been found in several surveys carried out in different countries [13,14,15]. Generally, PBDs are lower in fat (<1.5%) and protein (<1%), while the amount of carbohydrates is similar to that in milk (3–5%). The exceptions are oat drinks, which contain considerably more carbohydrates (~7%), and soy drinks, which contain a protein content similar to that of milk. In consideration of its similar nutrient composition compared to cow’s milk and its use in meals, only soy drinks (SDs) fortified with Ca, vitamin A and D were included in the dairy food group by Dietary Guidelines for Americans [19].

As a result of the differences in the nutritional composition between CM and most PBDs, it is of interest to investigate the effects of the CM substitution with different types of PBDs on markers of human health. Based on these premises, the aim of this review is to systematically analyze and summarize the main findings from human intervention trials comparing the effects of CM and PBDs on health-related markers. Since the substitution of CM milk with PBDs is growing, this review responds to the urgent need to understand the effects of consuming plant-based drinks instead of cow’s milk in the short and long term.

## 2. Materials and Methods

### 2.1. Literature Search Strategy

A systematic literature search was conducted using two different academic digital databases, PubMed^®^ and Scopus. The search was performed in May 2022 and updated in July 2022. The following search terms were used for PubMed: (“milk” OR “dairy”) AND (“soy” OR “rice” OR “almond” OR “oat” OR “vegetal” OR “plant-based” OR “alternative” OR “substitute*” OR “non-bovine”) AND (“human*” OR “person*”) AND (“intervention” OR “study” OR “trial”).

We used the Scopus database to search for articles, short surveys and conference papers in English in which the terms (“milk” OR “dairy product*”) AND (“soy” OR “rice” OR “almond” OR “oat” OR “vegetal” OR “plant-based” OR “alternative” OR “substitute*” OR “non-bovine”) AND (“human*” OR “person*”) AND (“intervention” OR “study” OR “trial”) were mentioned in the title, abstract or keywords. The search was performed for the period between 2000 and 29 July 2022. Reference lists of relevant manuscripts and reviews were examined for any possible unidentified studies.

The protocol was registered in PROSPERO (code: CRD42022319842).

### 2.2. Study Selection

Studies were considered eligible if they investigated the effects of consuming plant-based drinks compared with cow’s milk consumption on health-related parameters in humans aged 18 years and over. To be included, studies had to be human intervention trials evaluating the effects of acute or chronic intake. There were restrictions pertaining to age (≥18 years) but not to other characteristics of study participants (e.g., health condition, BMI, gender and education).

Studies were considered eligible if they were published in English.

Given these criteria, intervention studies were excluded if the target population was aged <18 years; they tested a plant-based protein isolate or protein blends; or the investigated outcomes differed from health- or disease-related markers (e.g., absorption of nutrients).

A more detailed list of eligibility criteria, developed by following the PICOS (Population, Intervention, Comparison, Outcome, Study Design) format, is shown in Table 1.

Two independent authors (P.B. and V.V.) conducted the study selection and evaluated the eligibility of the clinical trials. Disagreements were solved by consulting a third author (D.M.).

### 2.3. Data Extraction and Presentation

The following data were extracted from each study: name of the first author and year; study design; sample size and characteristics of enrolled subjects; location; PBD intervention; CM intervention; outcome variables; and results of the comparison between PBD and CM on health-related parameters.

## 3. Results

### 3.1. Study Selection

**Figure 1:**
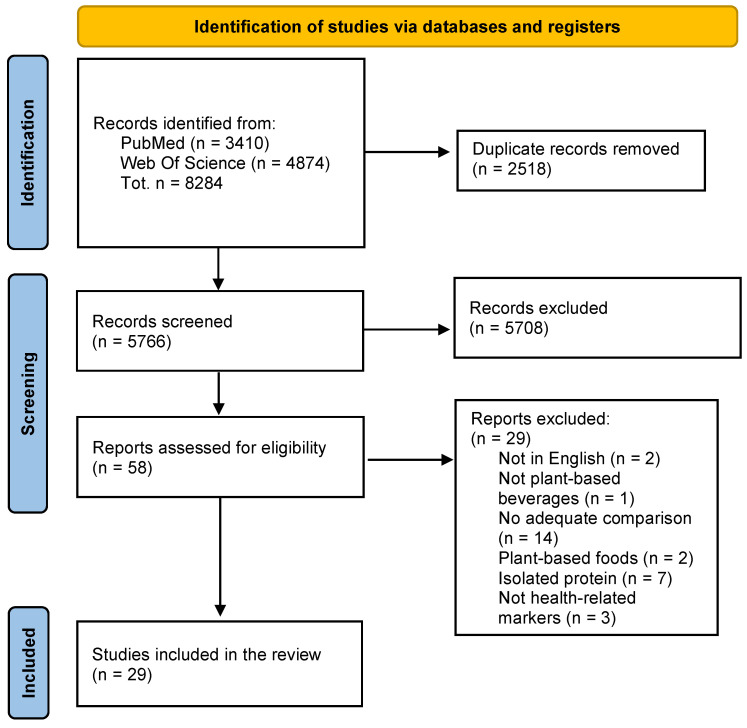
PRISMA flow chart of the systematic review literature search.

As reported in the PRISMA diagram (Figure 1), a total of 8284 records were identified from the database searches (PubMed^®^ and Scopus). After removing 2518 duplicate articles, 5766 studies were screened, and 5708 were excluded based on the title and abstract. A total of 58 eligible records went through the full-text screening process, and 29 studies were excluded because they did not meet the inclusion criteria. At the end of the selection process, 29 papers were included in the qualitative analysis; 27 studies evaluated the effect of a soy drink (SD) compared with CM [20,21,22,23,24,25,26,27,28,29,30,31,32,33,34,35,36,37,38,39,40,41,42,43,44,45,46], 1 of which also evaluated the effects of an almond drink (AD) [43], and 1 of which included two trials [29]. Two compared the health-related effects following rice drink (RD) and CM consumption [47,48]. Since studies comparing SD and CM were much more numerous than those comparing other PBDs, results were reported according to the type of PBD.

Finally, five trials compared the effects of CM not only with a PBD but also with other treatments (e.g., water or control diet without CM and PBD). In these cases, only the comparison between the PBD and CM was reported [33,35,42,43,44].

**Figure 2:**
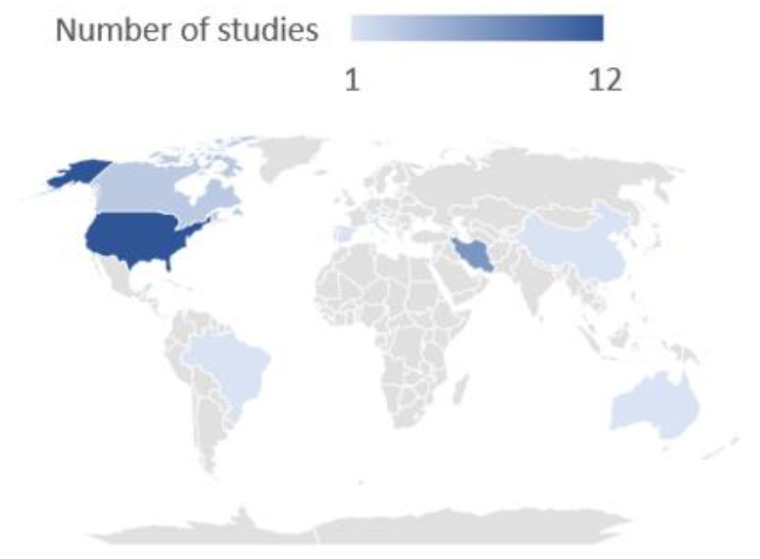
Geographical distribution of all the studies included in this review. Blue indicates the number of studies performed in the different countries: the darker the blue, the higher the number of studies conducted.

The number of studies performed in different countries is reported in Figure 2, which shows the countries where all the articles included in this review were performed. The highest number of studies (*n* = 12) was performed in the United States, followed by Iran (*n* = 7), Canada (*n* = 2) and Singapore (*n* = 2). Australia, Brazil, China, Italy, Japan and Spain contributed one study each.

#### 3.1.1. Study Design and Interventions

The main characteristics of the studies evaluating the effects of the acute intake (i.e., a single meal) [42,43,44,45,47,48] and long-term intake [1,2,3,4,5,6,7,8,9,10,11,12,13,14,15,16,17,18,19,20,21,22,23,24,25,26,27,28,29,30,31,32,33,34,35,36,37,38,39,40,41,46] of PBDs compared to CM are reported in Table 2a,b. From the 29 included studies, a total of 30 unique trials were available for detailed analysis since one of the publications reported two separate dietary interventions with SD [29].

The most investigated PBD was SD, with 27 studies reporting 28 unique trials that assessed its effects on health-related parameters compared to CM, among which 4 were single-meal studies. One trial out of twenty-eight also evaluated the effects of AD consumption [43], while two compared CM with a rice drink (RD) [47,48], with both studies on RD being single-meal interventions.

Out of the 28 unique trials performed with SD, 15 had a crossover design. The washout period ranged from 1 week to ≥4 weeks; one intervention did not include a washout period [22]. The remaining trials (*n* = 13) followed a parallel design.

The nutritional profile of SD used in the intervention trials was different among the studies; three studies did not report the specific nutritional composition of soy drinks used [26,33,44]. One study reported only the isoflavone and protein contents of the SD used [30], while other studies reported only the protein content [42] or only the isoflavone content of SD [41]. Most of the included studies tested Ca-fortified SD. Regarding that, it is important to highlight that not all studies reported the Ca content; in the studies reporting this information, the Ca content in 100 mL of SD ranged from 9 mg [22] to 140 mg [34].

In addition to traditional SD, studies also evaluated the effects of low-glycinin SD [34], SD produced using isolate soy protein [25], low-fat plant-sterol-enriched SD and moderate-fat plant-sterol-enriched SD [29].

The amount of SD tested ranged from 200 mL/d [46] to 1 L/d [20,22], and the treatment periods ranged from one meal [42,43,44,45] to 18 months of regular intake [35]; a large number of dietary interventions lasted 4 weeks (*n* = 12).

In most of the studies (*n* = 18), the CM used in the comparison with SD was reduced-fat milk, while only three studies used whole-fat milk [35,45,46]. In three studies, skim milk was used [20,22,26]. The remaining three studies did not report any information about the fat content of cow’s milk used [21,32,42].

The two studies comparing rice drinks with CM were both RCTs with a crossover design and a washout period of 1 week [48] or 1 week for men and 1 month for women to consider changes due to the menstrual cycle [47]. The first study evaluated the effect of one meal of a rice drink in a dose-dependent manner (473 mL and 946 mL) [48], while the second examined the effect of the acute intake of a rice drink (475 mL) [47]. In both studies, the CM used was low-fat.

Finally, the only RCT carried out on AD had a crossover design (washout period ≥ 1 week) and evaluated the different effects on health-related parameters following CM (250 mL) or AD (250 mL) acute intake [43].

#### 3.1.2. Subject Characteristics

A total of 1056 subjects were involved in the 28 trials testing SD in comparison with CM. In 12 trials, the target population was represented by healthy adults, while 5 trials examined the effects of SD on subjects with hypercholesterolemia. In seven studies, the target population was represented by adults with risk factors: six with overweight or obesity [26,32,33,34,36,38] and one with mild-to-moderate essential hypertension [20]. Two studies assessed the effects of SD consumption on cardiorenal biomarkers in type 2 diabetic patients with nephropathy [37,40], while two others did not report any information about the health status of subjects involved in the trials [30,31,32,33,34,35].

The mean BMI ranged from a minimum of 20.3 kg/m^2^ [45] to a maximum of 35.0 kg/m^2^ [48]. One study reported only the BMI ranges of the subjects recruited [42], while another study reported only the mean age [46]. One study did not report any data [20].

The mean age of the subjects ranged from a minimum of 22 years [32,45] to a maximum of 59 years [21]. Two studies reported the age ranges of the participants recruited without specifying the average [24,44].

In 14 studies, the dietary intervention was carried out only on women [22,24,26,27,28,30,31,32,33,35,36,38,39,41]; of these trials, 9 focused on postmenopausal women [23,24,26,28,30,31,35,39,41]. Three studies investigated the effects of the SD intervention compared with CM in men [34,42,44], while in ten studies, the target population included both men and women [20,21,22,25,29,37,40,43,45,46].

Among the two studies comparing the effects of CM and RD, one focused on healthy adults [48], and the other focused on subjects with metabolic syndrome [47]. In these studies, a total of 30 subjects were involved. The mean BMI ranged from 26.0 kg/m^2^ to 35.0 kg/m^2^, while the mean age ranged from 55 to 61 years. Finally, in the study assessing the effect of AD, the target population was healthy adults with a mean age of 23.0 years and a mean BMI of 22.3 kg/m^2^ [43].

**Figure 3:**
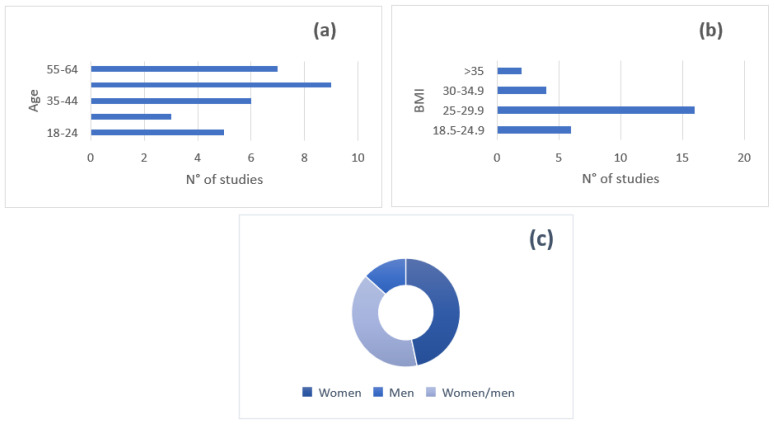
Characteristics of target populations of the trials included in the review: (**a**) age (*n* = 30); (**b**) range of BMI (*n* = 28 since two studies did not report these data); (**c**) gender (*n* = 30).

The overall characteristics of the target populations of all studies included in the review are shown in Figure 3.

### 3.2. Main Findings Derived from Comparisons between SD and CM

The main outcomes analyzed in the studies included in this review were anthropometric parameters (*n* = 13), markers of inflammation and/or oxidative stress (*n* = 7), lipid profile (*n* = 8), blood pressure (*n* = 4), and glucose and insulin levels (*n* = 6), while only one study focused on a marker of bone health [35] or other markers. Since the studies often analyzed more than one parameter, the total number of variables is higher than the number of studies.

In the following section, the results of the studies comparing the effects of SD and CM on health-related markers are specifically reported.

#### 3.2.1. Comparison between SD and CM Effects on Markers of Bone Health

Gui et al. conducted a parallel RCT to evaluate bone mineral density (BMD) in 141 postmenopausal Chinese women without osteoporosis. The participants were randomly assigned to consume 250 mg of Ca in CM (250 mL/d) or in Ca-fortified SD (250 mL/d) daily for 18 months. A downward trend or fluctuations in the lumbar spine’s BMD were found at 6 and 12 months in both intervention groups; no significant effects on the BMD of the lumbar spine at any site were found. The mean rate of gain of BMD in the hip and femoral neck at 18 months was significantly higher for the CM group compared to the baseline. The change in lumbar BMD was not significant between groups [35].

#### 3.2.2. Comparison between SD and CM Effects on Anthropometric Parameters

Among the 28 studies, 13 compared the effects of SD and CM consumption on anthropometric parameters. However, only two studies considered them primary endpoints [26,33].

The first study was an 8-week, prospective, parallel, randomized, controlled trial in which 14 women with overweight or obesity on a 500 kcal/d deficit diet were randomly assigned to the SD group (720 mL/d SD with soy protein supplement added for the equivalent protein intake of CM) or to the skim CM group (720 mL/d CM). The nutritional composition of SD and skim CM was not reported. After both interventions, a significant mitigation of the loss of fat-free mass and a significant reduction in weight, body fat percent and abdominal circumference were observed. However, these parameters did not differ significantly between groups [26].

The second trial was performed on 85 healthy overweight or obese premenopausal women who were randomized to one of the following dietary interventions for 8 weeks: (1) a CM diet providing a 500 kcal/d deficit containing three servings of low-fat milk or (2) an SD diet providing a 500 kcal/d deficit and containing three servings of Ca-fortified SD. After both interventions, a significant reduction in body weight (BW), BMI, waist circumference (WC), waist-to-hip ratio (WHR), body fat mass (BFM) and percent body fat (%BF) was observed. After adjustment for baseline values, changes in BW and BMI were significantly different between the two interventions, with significantly greater changes in the CM group than in the SD group [33].

In two other crossover clinical trials, overweight and obese female subjects on a weight-reducing diet were randomly assigned to consume 240 mL/d of CM or SD (containing 40 mg of Ca in 100 mL), and the effect on BW was analyzed [32,36]. In the first study, the intervention lasted 6 weeks, while in the second trial, the intervention was shorter (4 weeks). At the end of both trials, no significant differences in BW between the intervention groups were recorded [32,36]. In addition, Azadbakht et al. also analyzed WC and hip circumference, without finding any significant changes [32]. This is in line with results from Keshavarz et al., who recorded HR, BMI and WC and did not report any significant differences between the two intervention groups, except for a significant reduction in WC after SD compared to CM [36].

Three crossover trials assessed the effects of 4 weeks of the daily consumption of 240 mL of SD providing 96 mg of Ca on BW when compared to low-fat CM consumption (240 mL/d) in a female cohort [37,38,40]. The first trial was conducted on a target group composed of 24 non-menopausal overweight and obese women following a reduced-energy diet (with a deficit from 200 to 500 kcal/d) [37], while the target group of the other two trials was represented by 25 patients with diabetic nephropathy following a diet for nephropathy [38,40]. In all three studies, statistical analyses showed no significant differences in percent changes in BW after the SD period compared to the CM period [37,38,40].

The consumption of 3 servings/d of SD or low-fat CM for 4 weeks in postmenopausal women did not lead to any significant BW changes throughout the study [28,30,31].

BW changes were also analyzed in a 4-week, double-blind, crossover study by Sirtori et al. in 20 patients with type II hypercholesterolemia after the consumption of SD providing 25 g/d of protein or after the consumption of identically formulated CM (500 mL/d). Again, no significant BW changes were observed during the intervention [21].

Two studies compared the effects of SD and CM on BMI, and neither of them found any significant changes over the course of the interventions [22,23]. The first was a double-blind, randomized trial in which 60 outpatients with primary hypercholesterolemia following a lipid-lowering diet for at least 6 weeks were randomly assigned to consume 1 L/d of SD (containing 9.1 mg of Ca in 100 mL) or no-fat CM for 6 weeks [22]. The second one assessed the effects of 706 mL/d of Ca-fortified SD containing 71.6 mg of isoflavones plus a placebo supplement with 706 mL/d of low-fat CM with or without 70 mg of isoflavones in a supplement on 52 postmenopausal women in a 16-week, parallel trial [23].

#### 3.2.3. Comparison between SD and CM Effects on Markers of Inflammation and Oxidative Stress

A total of seven studies evaluated the effects of SD and CM on inflammation markers and/or markers of oxidative stress [22,23,28,30,37,38,39].

Nourieh et al. evaluated the effects of 240 mL/d of SD (40 mg Ca/100 mL of SD) or reduced-fat CM on two markers of inflammation, high-sensitivity C-reactive protein (hs-CRP) and interleukin 6 (IL-6). Compared to the baseline, a non-significant reduction in hs-CRP and a non-significant increase in IL-6 was observed following the consumption of SD, while IL-6 and hs-CRP increased in the CM group. However, this study did not find any significant changes in the parameters analyzed between the two treatments; only a marginal difference in IL-6 levels was observed between the two groups [38].

Miraghajani et al. evaluated the effects of 240 mL/d of SD (40 mg Ca/100 mL of SD) or reduced-fat CM on IL-6, TNF-α, hs-CRP and MDA. The analysis showed that there were no significant percent changes in these markers after the two intervention periods. There was only a reduction close to significance in hs-CRP levels after SD consumption compared to CM [37].

In addition, two RCTs with a parallel design evaluated both markers of inflammation and oxidative stress [23,30]. In the first trial, Ryan-Borchers et al. assessed the effects of soy isoflavones in Ca-fortified SD (127.5 mg Ca/100 mL) and in a supplement form on immune variables, including lymphocyte subsets, cytokine production (IFN-γ, TNF-α, IL-2), CRP, lipid peroxidation (8-Isoprostane) and DNA damage (8-hydroxy-2-deoxy-guanosine (8-OHdG)) in 52 postmenopausal women. The women enrolled were randomly assigned to the control group (706 mL/d low-fat CM plus a placebo supplement for 16 weeks), SD group (706 mL/d of Ca-fortified SD containing 71.6 mg isoflavones plus a placebo supplement for 16 weeks) and supplement group (706 mL/d of low-fat CM plus 70 mg of isoflavones in a supplement form for 16 weeks). After 16 weeks, the lymphocyte subpopulations did not significantly differ among the three experimental groups, except for B-cell populations, which were higher in the soy isoflavone groups (SD and supplemental form) than in the control group. Finally, a significant reduction in plasma 8-OHdG concentration was observed among women consuming isoflavones (SD and CM group) compared to women in the control group. Significant effects of group, week and group × week interaction on plasma 8-OHdG were also observed [23].

In the second trial, the consumption of 3 servings/d of vanilla SD was compared with the consumption of 3 servings/d of reduced-fat CM; parameters of systemic inflammation (TNF-α, IL-1β and IL-6) and the antioxidant defense system (SOD, GPx and COX-2) were measured before and after the enrolled women performed an eccentric exercise bout. The results of this study showed that the intervention periods did not significantly affect any markers of inflammation or the antioxidant defense system; however, it showed a significant group-by-time effect on plasma TNF-α in the CM group, with TNF-α values that increased post-supplementation and then decreased in the postexercise period [30].

Beavers et al. determined the effects of vanilla SD consumption (732 mL/d) compared with reduced-fat CM (709.8 mL/d) for 4 weeks on plasma markers of inflammation (TNF-α, IL-6, IL-1β) and oxidative stress (superoxide dismutase (SOD), glutathione peroxidase (GPx), cyclooxygenase-2 (COX-2)) in 31 postmenopausal women. This RCT with a parallel design showed no significant differences between the intervention groups, although there was an increase close to significance in TNF-α values after the consumption of CM compared to SD [28].

Serra et al. conducted a parallel RCT on 31 postmenopausal women to assess the effects of SD compared to reduced-fat CM on the expression of inflammation-responsive (TNF-α, IL-1β, IL-6) and proteolytic (calpain 1, calpain 2, ubiquitin, E2, atrogin-1, muRF-1) genes in skeletal muscle. These parameters were measured before and after the treatment and after the women performed a downhill run. After consuming three servings of SD or CM each day for 4 weeks, group effects were observed for TNF-α and atrogin-1 from T1 to T2; the concentration of TNF-α was greater with SD, while atrogin-1 was greater with CM (although it was just a trend). Significant main effects of time were reported for IL-1β, IL-6, calpain 2 and atrogin-1 mRNA. However, this trial did not show a significant group-by-time interaction for any of the markers analyzed [39].

Finally, Bricarello et al. evaluated the effect of 1 L/d of SD (containing 9.1 mg of Ca in 100 mL) compared to skim CM for 6 weeks on lipid peroxidation estimated by plasma thiobarbituric-reactive substances, which decreased significantly after SD compared to the CM intervention [22].

#### 3.2.4. Comparison between SD and CM Effects on Lipid Profile

Eight crossover trials from seven unique publications compared the effects of SD on lipid profiles with the CM intervention, using CM with different fat contents. Five of these interventions reported a significant reduction in low-density lipoprotein cholesterol (LDL-C) after SD consumption compared with CM consumption [22,25,29,38]. Specifically, Bricarello et al. showed a significant reduction in LDL-C levels and a significant increase in high-density lipoprotein cholesterol (HDL-C) levels after the SD intervention (1 L/d) compared with baseline values and skim CM consumption (1 L/d). Conversely, no significant effects of SD (providing 9.1 mg of Ca per 100 mL) in comparison with baseline and skim CM on total cholesterol (TC) and triacylglycerol plasma levels were observed [22].

Gardner et al. compared the effects on the plasma lipid profile of two SDs, one produced with whole soybeans (WB) and the other using soy protein isolate (SPI), with low-fat CM. The dietary intervention, composed of an amount of the assigned drink standardized to yield 25 g protein/d for 4 weeks, was followed by 28 hypercholesterolemic adults. The interventions with both WB and SPI promoted a significant decrease in LDL-C concentrations relative to CM, while no significant differences among groups for HDL-C and triacylglycerols were detected. A modest lowering effect of WB and SPI on the LDL-C/HDL-C ratio was observed compared to the CM group. The effects did not differ between different types of SD or between equol and non-equol producers [25].

Nourieh et al. evaluated the effects of 240 mL/d consumption of SD (containing 40 mg of Ca in 100 mL) for 4 weeks in comparison with reduced-fat CM (240 mL/d) on lipid profiles (triglycerides (TG), HDL-C, LDL-C, TC) among 24 non-menopausal overweight and obese female adults through a crossover randomized clinical trial with a washout period of 2 weeks. There were no significant changes regarding serum lipid concentrations (TG and HDL-C) at the end of the study, although the results for TC were marginally significant. A significant reduction in serum LDL-C following the SD period was observed compared to the CM period [38].

Rideout et al. conducted two separate 4-week crossover RCTs to evaluate the cholesterol-lowering efficacy of low-fat (2 g/serving) plant sterol (PS)-enriched SD on 33 normocholesterolemic subjects (study 1) or moderate-fat (3.5 g/serving) PS-enriched SD on 23 hypercholesterolemic subjects (study 2) in comparison with 1% fat CM. Both low- and moderate-fat PS-enriched SDs significantly reduced TC (by 10% in the first study), LDL-C (by 13%), and LDL/HDL-C and TC/HDL ratios compared to CM. There were no treatment effects on TG and HDL-C levels between the low-fat PS-enriched SD and the CM interventions. Conversely, a significant reduction in TG was observed after the consumption of moderate-fat PS-enriched SD compared to CM. In the first study, further analyses conducted on a subgroup of participants with initial LDL-C concentrations > 3.4 mmol/L showed a 9.5% reduction in plasma TC concentrations and a 12% reduction in LDL-C concentrations compared to controls. A significant reduction in cholesterol absorption in response to the consumption of PS-enriched SD in comparison to 1% CM was observed; conversely, no significant differences in cholesterol synthesis or HDL-C concentrations were observed between moderate-fat SD and the control groups [29].

A 4-week crossover RCT on 29 type 2 diabetic patients with nephropathy tested the effects on lipid profiles (serum TC, TG, HDL-C, LDL-C) of a diet containing 240 mL of Ca-fortified SD (40 mg of Ca in 100 mL of SD) or 240 mL/d of low-fat CM separated by a 2-week washout period. At the end of the trial, a significant reduction in serum TC and TG was observed in the SD compared to the CM intervention, but not after adjustment for carbohydrate intake. Moreover, no significant changes in other lipid parameters between the two intervention periods were found [40].

Similarly, no significant differences in plasma LDL-C or TC after the consumption of SD in comparison with an identically formulated CM were found in the double-blind crossover study by Sirtori et al. analyzing the impact on cholesterolemia. The researchers found only small reductions in TC and LDL-C, not reaching statistical significance, when the SD was given during the second period [21].

Finally, a parallel RCT conducted on 32 postmenopausal women showed that after 4 weeks of dietary interventions (3 servings/d of vanilla SD or reduced-fat CM), the levels of TC, TG, LDL-C, HDL-C and TC did not significantly change from the baseline or between interventions. A further analysis was conducted on those individuals with dyslipidemia without revealing any significant changes in TC, TG, LDL-C or HDL-C from the baseline or between treatments [31].

#### 3.2.5. Comparison between SD and CM Effects on Blood Pressure

Four studies specifically analyzed the effects of SD compared to CM consumption on blood pressure [20,32,36,40]; in three of them, 100 mL of SD provided 40 mg of Ca [32,36,40], while in one study, the Ca content of SD was 60 mg per 100 mL [20]. Although the interventions were different among the four studies, three of them showed that SD consumption led to a significant reduction in blood pressure compared to the CM intervention [20,32,40]; however, Keshavarz et al. did not report any significant differences between the two intervention groups [36].

In one crossover clinical trial, the consumption of SD led to a significant reduction in diastolic blood pressure (DBP) and systolic blood pressure (SBP) values compared to CM consumption [32]. In another crossover trial, the SD intervention resulted in a significant reduction in SBP compared with the consumption of reduced-fat CM; this change remained significant even after adjustment for carbohydrate intake [40].

Finally, an RCT with a parallel design assessing blood pressure values after the consumption of 1 L/d of SD or reduced-fat CM for 3 months was conducted on subjects with mild-to-moderate hypertension. In both groups, DBP, SBP and mean blood pressure showed a reduction after the treatments; however, the reduction in these values was significant and greater after the consumption of SD compared to CM. Furthermore, a significant negative correlation between the reduction in DBP and urinary genistein excretion and a tendency for a negative correlation between urinary equol excretion and a decrease in SBP were noted [20].

#### 3.2.6. Comparison between SD and CM Effects on Glucose and Insulin Levels

A total of six studies compared the effects of SD and CM consumption on glucose and insulin levels [25,36,40,43,44,45], among which three looked at the acute intake (i.e., single meal) [43,44,45] and three looked at the long-term intake [25,36,40] of the given products.

Regarding the former, Sun et al. conducted a randomized crossover trial to evaluate the effects of dietary interventions (322 mL of CM and 322 mL of sweetened SD) consumed before (preload) or together with (co-ingestion) white wheat bread on postprandial blood glucose, insulin and gastric emptying in healthy male participants. The composition of both drinks was not reported. The results revealed no significant differences in blood glucose concentrations after the co-ingestion of SD and CM, while a significant increase with an SD compared to a CM preload (at 30, 45 and 60 min) was found. At 30 and 45 min, preloading with SD was also associated with higher insulin responses than preloading with CM [44].

Sakuma et al. showed that serum insulin and plasma glucose levels did not differ significantly between SD and CM consumers. This was a randomized crossover trial conducted on 10 healthy subjects consuming three test meals composed of (1) unadjusted SD containing 12.5 mg of Ca in 100 mL, wheat bread, strawberry jam and soft margarine; (2) Ca-fortified SD containing 117 mg of Ca in 100 mL, wheat bread, strawberry jam and soft margarine; (3) whole-fat milk, water, wheat bread, strawberry jam and soft margarine [45].

Another randomized crossover trial tested the effects of 250 mL of SD or 1% fat milk with 54 g of breakfast cereals. After 2 h, participants were fed an ad libitum meal (three varieties of pizza), and satiety, insulin and glucose parameters were measured. A time × treatment interaction was documented. In detail, a lower blood glucose peak was found at 30 min following the consumption of SD compared to the CM treatment. Post-treatment blood glucose was lower after SD consumption compared to CM consumption, while no significant differences were observed in post-meal or cumulative values between the two treatments. After 30–120 min, changes in insulin concentration from the baseline were affected by the treatment, time and time-by-treatment interaction. CM led to a higher insulin concentration compared to SD, while no significant differences were observed between post-meal and cumulative values. In the post-meal period, mean insulin changes from the baseline were affected by the treatment and time but with no time-by-treatment interaction. Following SD consumption, a significantly lower post-treatment value of AUC for insulin was observed compared to the CM intervention. No other differences between post-meal and cumulative insulin AUCs were observed. There were no differences between treatments in post-treatment, post-meal or cumulative ratios of blood glucose to insulin AUC or in the ratio of blood glucose to insulin changes from the baseline at 30 min [43].

Among studies evaluating the effects of the long-term substitution of CM with SD, a crossover study conducted on patients with type 2 diabetes and nephropathy showed that insulin and hemoglobin A1C did not differ between the two dietary interventions containing 240 mL of Ca-fortified SD (40 mg of Ca in 100 mL of SD) or 240 mL/d of low-fat CM for 4 weeks [40]. The same results were observed in another crossover trial in which the intervention included two SDs, one produced with whole soybeans (WB) and the other with soy protein isolate (SPI), and one reduced-fat CM. The WB drink contained 13.2 mg aglycone equivalents of total isoflavones in 100 mL, while 4.7 mg of aglycone equivalents of total isoflavones was provided by 100 mL of the SPI drink. This trial did not find any significant differences in insulin and glucose responses among the different interventions [25].

Finally, Keshavarz et al. showed that glycemic control indices did not change significantly after the consumption of 240 mL/d of SD containing 40 mg of Ca in 100 mL compared to the consumption of 240 mL/d of reduced-fat CM [36].

#### 3.2.7. Comparison between SD and CM Effects on Other Health-Related Parameters

The effects of SD and CM on other health-related parameters have been studied only in a limited number of trials, as reported below for each variable considered.

Appetite: Law et al. showed that both reduced-fat CM and SD treatments equally suppressed appetite with no differences between them. It was also observed that energy intake was lower after CM than SD [43].

Coagulation: Two studies analyzed fibrinogen, and they did not find any significant differences between intervention groups [36,37]. In addition, Miraghajani et al. evaluated D-dimer levels, and they reported a significant reduction in its levels among all participants; however, after adjustment for carbohydrate intake, this result lost statistical significance. However, differences in percent changes in D-dimer levels after the consumption of reduced-fat CM and SD containing 40 mg of Ca in 100 mL remained significant, even after adjustment for carbohydrate intake [37].

Cognitive function: Fournier et al. investigated whether soy isoflavones could have a beneficial role in cognitive function in healthy, postmenopausal women. They conducted a 16-week, double-blind, placebo-controlled, parallel trial in which 79 postmenopausal women were assigned to one of three intervention groups: the control group (706 mL/d reduced-fat CM + placebo supplement); SD group (706 mL/d Ca-fortified SD + placebo supplement); or CM group (reduced-fat 706 mL/d CM + isoflavone supplement). The Ca-fortified SD provided 127.5 mg of Ca per 100 mL. Before and after the intervention, cognitive function was evaluated, and a reduction in verbal working memory (Digit Ordering Task) was noted in SD groups compared to the control and CM groups. Conversely, no significant variations were observed in selective attention (Stroop task), visual long-term memory (pattern recognition), short-term visuospatial memory (Benton Visual Retention Test), visuospatial working memory (Color Match task), verbal memory span (Forward Digit Span) or spatial memory span (Corsi Block-Tapping) across groups. In terms of reaction time, improvements in Color Match performance in the test were significantly different from zero in all the groups relative to the baseline. Finally, a practice effect was noted for the pattern recognition task since improvements in accuracy in the test were significantly different from zero relative to the baseline in all the intervention groups [24].

Gut microbiota composition: In a 3-month, randomized, parallel, double-blind trial, Fernandez-Raudales et al. compared the effects of 500 mL/d of low-glycinin SD (LGS, 49.5% β-conglycinin/6% glycinin), conventional SD (SD, 26.5% β-conglycinin/38.7% glycinin) and reduced-fat CM on the intestinal microbiome of 64 overweight and obese men. Both the tested SDs were fortified with Ca (139.8 mg of Ca in 100 mL of LGS and 132 mg of Ca in 100 mL of conventional SD). After the treatments, the total bacteria, *Bacteroides-Prevotella*, *Bifidobacterium* and *Lactobacillus* did not significantly change among groups. A significant increase in the total bacteria over time was observed within the intervention groups; in addition, a significant increase in *Bacteroides-Prevotella* in LGS groups and *Lactobacillus* in CM groups was noted. Conversely, the consumption of LGS and SD led to a significant decrease in *Bifidobacterium*, Firmicutes and the Firmicutes-to-Bacteroidetes ratio. The relative abundance of Bacteroidetes increased in both SD groups and decreased in the CM group, although these changes only approached statistical significance. All the interventions resulted in a significant increase in the relative abundance of members in the phylum Proteobacteria and a decrease in bacterial diversity and bacterial richness. A treatment effect approaching statistical significance was observed for the bacterial community between CM and SD groups. After SD, *Roseburia* tended to decrease, while *Prevotella* tended to increase; in the CM group, the opposite trend was observed. After the consumption of LGS, *Faecalibacterium* tended to increase. For *Lactobacillus*, the indicator score was decreased in LGS and SD groups, while it increased in the CM group [34].

Hormones, amino acid profile, serum/urine Ca and phosphorus levels: Sun et al. conducted a randomized, crossover study involving three experimental single-meal interventions with a washout period of 1 week on 12 healthy men. The aim of this study was to compare the effects of SD and CM consumption on incretin hormone secretion after the consumption of white bread and isovolumetric amounts of SD, CM or water (322 mL); the content of protein was the same (12 g in 322 mL) in SD and CM. For both postprandial glucagon-like peptide-1 (GLP-1) and glucose-dependent insulinotropic polypeptide (GIP), a significant main effect of the drink type was observed. The ingestion of CM led to a higher active GLP-1 concentration compared with SD; conversely, postprandial GIP concentrations were greater after the SD intervention compared to CM. The peak of GIP was much later after CM compared to SD consumption. Positive correlations were observed for the first 30 min between modifications in alanine and arginine and the corresponding change in GIP. A non-significant positive correlation was found between GLP-1 and valine, isoleucine, leucine and branched-chain amino acids (BCAA). CM treatment led to an increase in BCAA compared to SD [42].

In a randomized crossover trial, after the ingestion of each meal containing (1) unadjusted SD, (2) Ca-fortified SD or (3) CM, serum intact parathyroid hormone (S-PTH) levels of young men first showed a drop, followed by a progressive rise. After the ingestion of the SD meal, postprandial S-PTH was significantly higher than that following the CM meal and the SD + Ca meal at 30, 60, 120, 240 and 360 min; in addition, the authors observed a reduction in serum Ca levels compared to CM meal and SD + Ca meal ingestion. Finally, an increase in serum phosphorus levels was observed after the consumption of the CM meal compared to the SD meal and SD + Ca meal. Among the meal groups, no significant changes were noted in urine phosphorus or Ca levels [45].

Finally, a 16-week, parallel, placebo-controlled trial evaluated the effects of soy isoflavones on thyroid-stimulating hormone (TSH) in 77 healthy postmenopausal women. The participants were randomly assigned to one of the following treatments: (1) 706 mL of reduced-fat CM/d and a placebo supplement (CM group), (2) 706 mL of Ca-fortified SD/d (127.5 mg of Ca in 100 mL) and a placebo supplement (71.6 ± 3.1 mg isoflavones/d) (SD group) or (3) 706 mL of reduced-fat CM/d and tablets with 70 mg of isoflavones. None of these interventions led to significant differences in TSH concentrations [27].

Kidney function: A 4-week RCT found that the replacement of CM consumption with SD had no effects on markers of kidney function. The comparison between the two treatments was carried out in terms of blood urea nitrogen, proteinuria, glomerular filtration rate, urine and serum creatinine [40].

Liver function: Keshavarz et al. found that the replacement of 240 mL/d of reduced-fat CM with SD, containing 40 mg of Ca in 100 mL, had no effects on alanine aminotransferase and aspartate aminotransferase levels [36].

Quality of life: An 8-month, randomized, parallel RCT aimed to assess the effects of SD (500 mL) in substitution for low-fat CM (500 mL) on quality-of-life-related parameters in 57 healthy postmenopausal women. The nutritional composition of SD was not available except for the isoflavone content (28.9 mg of genistein and 8.3 mg of daidzein in 100 mL of SD). Both groups also received 500 mg of Ca and vitamin D3 (200 IU). In both groups, the comparison between the pre-test and post-test scores revealed a significant mean score reduction in the vasomotor domain. The mean score of sexual domains did not decrease significantly in the SD group, while it increased significantly after the CM intervention. Furthermore, no significant changes from the baseline were observed in psychosocial and physical domains after either intervention. After the comparison of the percentage change in all the domains tested (vasomotor, psychosocial, physical and sexual), only the sexual domain score showed a significant increase after the CM compared to the SD intervention. There was a non-significant reduction in the mean scores of psychosocial and physical domains after the SD compared to the CM group [41].

Tooth enamel lesions: A double-blind, randomized, crossover study was conducted on eight healthy adults wearing a custom-made palatal appliance containing four enamel half-slabs with subsurface lesions. The participants were randomly assigned to consume 200 mL/d of full-cream milk or Ca-fortified SD (120 mg Ca/100 mL) for 15 days. A significant increase in the lesion depth after the exposure to SD and a significant decrease in the lesion depth following the consumption of CM were observed. In addition, a significant difference in the mineral content between the two interventions was documented; in fact, the mineral content decreased after the consumption of SD, while it increased following the CM intervention. This study also analyzed saliva Ca and fluoride levels post-drink consumption, and it was found that Ca and inorganic phosphate levels after CM consumption were significantly higher than those in the SD group; however, fluoride levels did not differ significantly [46].

### 3.3. Main Findings Derived from Comparisons between RD and CM

As mentioned above, both studies on RD included in the present review looked at the acute intake of RD compared to CM. In an RCT crossover trial, 11 healthy subjects were randomly assigned to consume 473 mL and 946 mL (two or four servings) of 1% CM or an RD (containing 126.8 mg of Ca in 100 mL) on four different days. The aim of the study was to evaluate the effects of these treatments on NO-dependent vasodilation (RBF, CV) and plasma insulin concentrations. Although the local heating plateau was not significantly different between the two treatments, the % NO-dependent vasodilation was significantly lower following CM ingestion compared to RD ingestion. The decreased NO-dependent vasodilation after the CM treatment was associated with a lower plasma insulin concentration. In fact, a significant reduction in the plasma insulin concentration following CM compared to RD intake was observed following both two and four servings of the products [48].

A further randomized crossover study analyzed the effect of the acute ingestion of 475 mL of low-fat milk compared to 435 mL of Ca-fortified RD (containing 119.1 mg of Ca in 100 mL) on endothelial vascular function, oxidative stress and NO bioavailability. Postprandial FMD was not affected by CM, while it decreased after RD. Regarding the other markers, glucose and malondialdehyde increased more in the trial with RD compared with CM, while arginine decreased after RD and increased after CM [47].

### 3.4. Main Findings Derived from the Comparison between AD and CM

In an RCT with a crossover design (washout period ≥ 1 week), 26 healthy adults were randomly assigned to consume non-isocaloric amounts (250 mL) of AD or 1% fat CM with cereals followed by an ad libitum meal 120 min later. In this study, subjective appetite, glucose and insulin were measured at baseline and at different time intervals.

The analysis of the effects of treatments on the measured variables showed the absence of any significant changes between CM and AD consumption. However, insulin and glucose were higher after CM consumption, but with no significant difference between treatments. The only significant difference was observed in the glucose/insulin iAUC ratio between post-treatment values after CM and AB. The glucose/insulin iAUC ratio was higher following the AD treatment compared to CM consumption [43].

## 4. Discussion

There is currently a wide-ranging debate on the actual effect of the substitution of CM drinks with plant-based ones, even though the consumption of plant-based dairy alternative drinks has been increasing, lowering the sales of dairy milk (e.g., in the United States −15% from 2012 to 2019) [49]. There are several reasons for this trend, as previously reported, including the increasing concerns about the impact of diet (particularly those rich in animal-based products) on planet health. Moreover, the negative public messages being spread related to CM, including those about the negative impact on human health and the increased risk of diseases such as cancer, have also probably been pushing consumers toward this transition [50].

Due to the increasing consumption of PBDs, which often completely substitute CM in the diets of several consumer groups, despite the different compositions of the two types of products, there is a growing interest in understanding the actual impact of this substitution on nutritional and health-related markers. Thus, the current review summarizes the “few” main findings available from intervention trials that evaluate the effects of substituting CM with PBDs on health-related markers.

We found that only 29 studies evaluated the effects on health-related markers following the substitution of CM with PBDs, and all the studies were performed on subjects over 18 years of age.

The limited number of studies were mainly focused on SD (*n* = 27), while the remaining studies evaluated the substitution of CM with RD (*n* = 2) and AD (*n* = 1). This is an interesting point considering that at least 20 different PBDs derived from cereals, legumes, nuts, pseudo-cereals and seeds are available on the market and widely consumed [6].

Surprisingly, we found only one study focusing on the impact of CM and a PBD on markers of bone health, such as BMD [35]. This is worthy of note since peak bone mass attainment is significantly influenced by nutrients such as Ca and protein; therefore, since dairy foods contain high-biological-value proteins and are the main sources of dietary Ca, their consumption is associated with optimal bone health throughout all stages of life [51,52]. In detail, the consumption of dairy products, including milk, has beneficial effects on bone mass accrual in children and adolescents and on bone turnover in young and older adults [52]. In fact, markers of bone health, such as bone mineral content (BMC) and BMD, were found to be lower in adults who had a low consumption of milk, and a reduced intake of milk in childhood was associated with a two-fold higher fracture risk [53]. At the same time, children who had avoided milk consumption and had not used food substitutes properly fortified with calcium were shown to have an increased risk for prepubertal bone fractures [54]. In older subjects, a higher consumption of milk and yogurt was associated with a lower risk of frailty [55]. Therefore, due to the recognized beneficial role of the consumption of milk and dairy products in bone health and the detrimental effect of their exclusion from the diet on outcomes related to bone health (i.e., fractures), the lack of intervention studies evaluating the impact of CM substitution with PBDs on these markers is undoubtedly noteworthy. This is even more relevant considering that the main nutritional differences between CM and non-fortified PBDs are related to Ca and vitamin D, which have a strong impact on bone health. For the above-mentioned reasons, it will be pivotal to perform additional studies exploring the impact of the replacement of animal-derived products [56,57], primarily on markers of bone health, which represents a major critical issue to be clarified.

Regarding the other variables, a thorough comparison of the findings of the different studies included in this review was difficult due to the high variability found. In fact, considering only the 27 studies investigating SD, high variability in terms of the doses and nutritional composition of PBDs (e.g., fortified or not fortified) and CM (e.g., skim, reduced or full-fat), study design (e.g., uncontrolled diets and free-living conditions), characteristics of test meals able to mask any possible changes in health -parameters (e.g., glucose/insulin changes), characteristics of the recruited subjects, duration and markers considered was found. Moreover, the effect on some specific markers, such as parathyroid hormone, alanine aminotransferase and aspartate aminotransferase levels, were investigated in a very limited number of studies, impeding our ability to draw any conclusions.

Even though the trials included are rather few and heterogeneous, the results of this systematic review seem to suggest a potential protective role of SD in the modulation of the lipid profile when compared to CM, which, however, has been shown to be associated with a decreased risk of CVD in observational studies [58]. In fact, five of the eight studies included showed that the consumption of SD compared to CM resulted in an LDL-C-lowering effect. Among soy components able to explain the lowering effect on LDL-C, it has been hypothesized that the α’ subfraction of the 7s globulin of soy protein may act through the upregulation of LDL receptors [59], while saponins can interact with bile acids, leading to the formation of mixed micelles, impairing cholesterol absorption [60]. Regarding isoflavones, which may enhance bile acid excretion and reduce cholesterol metabolism and the insulin-to-glucagon ratio [61], the data still seem to be uncertain and need further confirmation [25,62]. In addition, the LDL-C-lowering effect of SD could be related to decreased saturated fat (SFA) and cholesterol contents and to the higher polyunsaturated fat (PUFA) content of SD compared to CM, as hypothesized in one of the included studies [25].

Despite the positive effects of SD on LDL-C levels found in five studies, it is noteworthy that in two studies, the plasma-cholesterol-lowering effect might be explained because the SDs used were fortified with PSs. In fact, PSs are plant constituents chemically resembling cholesterol, and their lipid- and especially cholesterol-lowering effects are well known [63]. Furthermore, in another three studies, no significant differences were found in LDL-C levels between SD and CM interventions [21,31,40]. These contrasting results might be due to the lower intake of SD used in these three studies compared with those finding a significant effect. In addition, other potential confounding factors could be related to the use of different types of CM (skim in some cases and reduced-fat CM in others), as well as to the baseline cholesterol status of participants and the different isoflavone patterns of SDs used.

Among the four studies evaluating the effect of CM substitution with SD on blood pressure, three of them showed a greater reducing effect following SD consumption. The blood-pressure-lowering action of soy could be due to the higher content of arginine, a precursor of nitric oxide [20,64], and to the content of ACE-inhibitory peptides. Soy drinks, in fact, are a source of these peptides, which, by restricting the vasoconstrictor effects of angiotensin II and potentiating the vasodilatory effects of bradykinin, may exert a blood-pressure-lowering effect [65].

Once again, it is noteworthy that milk consumption has also been associated with lower BP, and higher dairy product consumption was associated with a reduced risk of hypertension in prospective cohort studies [66,67,68]. Similarly, convincing evidence of an association between total dairy consumption and a decreased risk of hypertension and a probable association with a decreased risk of elevated blood pressure was reported by Godos and coworkers, with the strongest evidence found for milk [58]. This effect may be attributed to the presence of minerals such as Ca and phosphorus and other components that may act through several mechanisms, such as the enhancement of renal sodium excretion, a reduction in intracellular Ca concentration and increased nitric oxide synthesis [67,69]. However, since saturated fatty acid content may decrease Ca and magnesium absorption, more studies comparing the effects of whole-fat and low-fat milk and dairy foods are needed.

Therefore, although the evidence from this review is somewhat equivocal, it seems to suggest that SD could positively affect blood pressure and the serum lipid profile, mostly in hypercholesteremic and overweight/obese subjects. These effects, if supported by future ad hoc studies in different target populations, could be of note since they are the main and modifiable drivers of CVD, the leading cause of premature mortality across the world [70,71,72].

Regarding other health-related outcomes, we did not find any interesting differences between the consumption of SD and CM. For instance, regarding anthropometric parameters, among the 13 studies, only 2 found a significant difference between the consumption of SD and CM. In detail, Faghih et al. reported a greater weight reduction after the consumption of SD compared to CM [33], while in the study of Keshavarz et al., a significant reduction in WC after SD compared to CM consumption was observed [36]. The remaining studies comparing the two different treatments did not find any significant modifications in anthropometric parameters.

Overall, taken together, the results discussed in the present review underline that the evidence of the beneficial effects of the substitution of CM with PBDs is still limited, especially for markers other than the lipid profile and blood pressure. This is true, for example, for variables related to the metabolic response and eating behavior, for which only a few results have been found, as well as for markers of oxidative stress and inflammation. In addition, the lack of differences in responses between the different treatments may be considered an interesting result.

However, differences in the characteristics of subjects included in the intervention trials could affect the results, compromising the possibility of extrapolating general recommendations. Therefore, future investigations should also explore the impact of these interventions in different target groups of the population, including the evaluation of how the different characteristics of the subjects (e.g., age, race/ethnicity, risk factors, health and socioeconomic status and other individual characteristics) may affect the findings.

Moreover, although the current review suggests a positive impact of CM substitution with SD on the lipid profile and blood pressure, the possible detrimental role of CM exclusion diets or substitution with non-Ca-fortified PBDs on bone health cannot be underestimated, despite the lack of current evidence on this topic derived from intervention studies; therefore, future studies should primarily investigate the effects of this substitution on markers of bone health.

Thus, based on current evidence, since CM is a key source of important nutrients for bone health (e.g., calcium and proteins), the total substitution of CM with PBDs requires the careful reading of food labels in order to select properly fortified PBDs. In detail, considering that only SDs fortified with Ca and vitamins A and D have been included in the dairy food group by Dietary Guidelines for Americans because of their similar nutrient composition [19], these PBDs should be preferred as possible substitutes for CM. In fact, the elimination of CM from the diet and its substitution with PBDs should be performed while paying especially close attention to the lower Ca content and the different protein quality and quantity found in some of these products [12,73]; at the same time, it would be of interest to investigate the impact of potential nutritional inadequacies in the long term when PBDs with different nutritional compositions compared to CM are chosen (e.g., not fortified with calcium), since these inadequacies may in turn negatively affect markers of human health, which have not been sufficiently investigated so far (e.g., the impact of bone turnover markers).

## Figures and Tables

**Table 1:** PICOS table for inclusion of studies.

Parameter	Inclusion Criteria
Population	Age > 18 years
Intervention	Dietary interventions evaluating the effect of PBD
Comparison	CM
Outcomes	Health and disease markers
Study design	Clinical trials

Legend: CM, cow’s milk; PBD, plant-based drink.

**Table 2:** Characteristics of the studies investigating the effects of (**a**) acute intake (i.e., single meal) and (**b**) long-term intake of cow’s milk compared with plant-based drinks.

(a) Acute Intake (i.e., Single Meal)						
Reference, Country	Study Design	Study Population ^a^	Soy Drink (SD) Intervention ^b^	Cow’s Milk (CM) Intervention ^b^	Health Outcome ^c^	Health-Related Findings ^c^
[42]	Postprandial, randomized, crossover, controlled trial (washout period 1 week)	20 healthy men Age: 21–26 years BMI: 18.3–24.4 kg/m^2^	322 mL of SD (containing approximately 12 g of soy protein) Composition: n.a.	322 mL of CM (containing approximately 12 g of milk protein) Composition: n.a.	Plasma total active GLP-1, GIP and plasma amino acid profile	↑ Postprandial active GLP-1 at 90, 120 min with CM vs. SD ↑ GIP with SD at 30, 60, 90 min vs. CM Positive correlations between changes in alanine and arginine and the corresponding change in GIP for the first 30 min ↑ Plasma BCAAs in CM vs. SD
[43]	Postprandial, randomized, non-blinded, crossover, controlled trial (washout period ≥ 1 week)	26 healthy adults (13 F, 13 M) Age: 23.0 ± 2.6 years BMI: 22.3 ± 1.5 kg/m^2^	250 mL of SD Composition per 100 mL: 3.2 g carbohydrate 1.6 g fat 2.8 g protein	250 mL of CM Composition per 100 mL: 4.8 g carbohydrate 1.2 g fat 3.6 g protein	Subjective appetite, insulin and GLU	Post-treatment blood GLU changes from baseline affected by treatment, time and a time-by-treatment interaction ↓ GLU peak at 30 min with SD vs. CM ↓ Post-treatment GLU with SD vs. CM Post-treatment changes in insulin concentrations from baseline affected by treatment, time and a time-by-treatment interaction ↑ Insulin concentrations post-treatment with CM vs. SD ↓ Post-treatment value of iAUCs for insulin with SD vs. CM ⟷ For appetite between CM vs. SD
[44]	Postprandial, randomized, crossover, non-blind trial (washout period 1 week)	12 healthy males Age: 21–26 years BMI: 21.5 ± 1.4 kg/m^2^	322 mL of sweetened SD Composition: n.a. The test meals were: (1) Preload SD + white bread consumed 30 min later (P-SD) (2) Co-ingestion of SD and bread (CI-SD) For the meal (SD + white bread): 50 g carbohydrate 6 g fat 17.6 g protein	322 mL of CM Composition: n.a. The test meals were: (1) Preload low-fat cow’s milk + white bread consumed 30 min later (P-CM) (2) Co-ingestion of low-fat CM with bread (CI-CM) For the meal (CM + white bread): 50 g carbohydrate 5.2 g fat 17.6 g protein	GLU, insulin, GI, II and gastric emptying	Blood GLU response affected by time, treatment and the interaction between time and treatment ↓ GLU with P-SD at 30–60 min vs. CI-SD ↑ GLU with P-SD at 120, 150 min vs. CI-SD ↓ GLU after test meal with P-CM at 15, 30 and 45 vs. CI-CM ↑ GLU with P-SD at 30, 45 and 60 vs. P-CM ↓ Peak of GLU response with preloading both drinks vs. their co-ingestion ↑ GLU iAUCs (30, 45, 60 min) P-SD vs. P-CM ↓ GLU iAUCs (from 30 to 120 min) P-SD vs. C-SD ↓ GLU iAUCs P-CM vs. C-CM at all time points ↓ GI of the preload drinks vs. co-ingestion drinks Plasma insulin response dependent on time, treatment and the interaction between time and treatment ↓ Insulin response with P-SD at 30, 60 min vs. CI-SD ↑ Insulin response with P-SD at 120, 210 min vs. CI-SD ↑ Insulin response with CI-CM at 30, 45 min vs. P-CM ↓ Insulin response with CI-CM at 120, 180 min vs. P-CM ↑ Insulin response with P-SD at 30, 45 min vs. P-CM ↓ Peak of insulin response and II with preloading with both drinks vs. their co-ingestion ↓ Insulin iAUCs with P-CM (between 30 and 150 min after the meal) vs. P-SD ↑ Insulin iAUCs at 45–120 min with CI-SD vs. P-SD ↑ II after P-SD vs. P-CM Gastric emptying affected by time, treatment, and the interaction between time and treatment ↑ Slow gastric emptying CI-SD at 15 min and P-CM at 120 min vs. P-SD
[45]	Postprandial, randomized, crossover trial (washout period 1 week)	10 healthy subjects (6 F, 4 M) Age: 22.4 ± 1.2 years BMI: 20.3 ± 6.3 kg/m^2^	(1) 335 g of SD Composition per 100 mL: 1.2 g carbohydrate 3.1 g fat 4.7 g protein 59.7 mg phosphorus 12.5 mg Ca 1.0 Na (2) 305 g of Ca-fortified SD composed of200 g of Ca-fortified SD + 105 g of SDComposition per 100 mL g of Ca-fortified SD: 4.5 g carbohydrate 2.3 g lipid 2.7 g protein 117 mg Ca 68.5 mg P 0 mg Na	225 g of CM Composition per 100 mL: 4.9 g carbohydrate 4.0 g fat 3.3 g protein 112 mg Ca 88.9 mg P 43.1 mg Na	Serum insulin, plasma GLU and intact PTH levels, serum and urine Pi and Ca	↑ S-PTH levels with SD meal at 30, 60, 120, 240, 360 vs. CM meal ↑ S-PTH levels with SD-Ca meal at 60, 120, 240, 360 vs. SD + Ca meal ↑ S-Pi levels with CM meal vs. SD meal (at 30, 60 and 120 min) and SD + Ca meal (at 60 and 120 min) ↓ S-Ca levels with SD meal vs. CM meal (at 60, 120, 240 and 360 min) and SD + Ca meal (at 30, 60, 120, 240 and 360 min) ⟷ For other parameters between CM vs. SD
**Reference**	**Study Design**	**Study Population ^a^**	**Rice Drink Intervention ^b^**	**Cow’s Milk Intervention ^b^**	**Health Outcome ^c^**	**Health-Related Findings ^c^**
[47]	Postprandial, randomized, double-blind, crossover trial (washout period 1 week for men, 1 month for women)	19 adults (5 F, 14 M) with metabolic syndrome Age: 28.5 ± 2.2 ^§^ years BMI: 35.0 ± 0.9 ^§^ kg/m^2^	435 mL of RD Composition per 100 mL: 9.2 g carbohydrate 0.9 g fat 0.2 g protein 119.1 mg Ca	475 mL of low-fat CM Composition per 100 mL: 5.1 g carbohydrate 1.1 g fat 3.4 g protein 128.4 mg Ca	FMD, plasma GLU, insulin and TGs, AUC_SR_, MDA, ADMA, ADMA:ARG ratio, fasting mean arterial pressure, heart rate, antioxidant status and oxidative/nitrative stress	↓ Postprandial FMD of the brachial artery with RD at 30, 60 min vs. baseline ↑ Time-matched FMD responses at 30 min with CM vs. RD ↑ FMD AUC_0–3h_ with CM vs. RD ↓ Main effect of time on maximal post-occlusion diameter with RD at 30 min vs. baseline ↓ AUC_SR_ with CM and RD at 30 min vs. baseline ↑ GLU (from 30 to 120 min) with RD vs. baseline ↑ GLU (30, 120, 150 min) with CM vs. baseline ↑ Insulin (from 30 to 150 min) with RD vs. baseline ↑ Insulin (from 30 to 120 min) with CM vs. baseline The increase in GLU (from 30 to 150 min) was larger during the RD compared to the CM trial ↑ ARG and ARG AUC_0–3 h_ with CM vs. RD ↑ ADMA at 30 min with RD and ↑ at 30–60 min with CM compared to baseline ↑ Time-matched ADMA concentrations at 90, 120 and 180 min with CM vs. RD ↑ ADMA/ARG ratio with CM at 30 min vs. baseline ↑ ADMA/ARG ratio with RD at 60–180 min vs. baseline ↑ ADMA/ARG AUC_0–3h_ with RD vs. CM
[48]	Postprandial, randomized, crossover, controlled trial (washout period ≥ 1 week)	11 healthy subjects (6 F, 5 M) Age: 61 ± 2 ^§^ years BMI: 26.1 ± 0.6 ^§^ kg/m^2^	(1) 473 mL (2 servings) of RD (2) 946 mL (4 servings) of RD Composition per 100 mL of SD: 9.7 g carbohydrate 0.8 g fat 0 g protein 126.8 mg Ca	(1) 473 mL (2 servings) of CM (2) 946 mL (4 servings) of CM Composition per 100 mL: 5.1 carbohydrate 1.1 g fat 3.4 g protein 126.8 mg Ca	NO-dependent vasodilation (RBF, CVC) and insulin	↓ %NO-dependent vasodilation with CM vs. RD ingestion ↓ Plasma insulin concentration with CM vs. RD for both the 2 and 4 servings; it was associated with a reduction in NO-dependent vasodilation
**(b) Long-Term Intake**						
**Reference, Country**	**Study Design**	**Study Population ^a^**	**Soy Drink (SD) Intervention ^b^**	**Cow’s Milk (CM) Intervention ^b^**	**Health Outcome ^c^**	**Health-Related Findings ^c^**
[20]	3-month, randomized, parallel, double-blind, controlled trial	40 adults (15 F, 25 M) with mild-to-moderate hypertension SD group (*n* = 20: 6 F, 14 M) Age: 47.5 ± 10.4 years BMI: n.a. CM group (*n* = 20: 9 F, 11 M) Age: 49.4 ± 10.8 years BMI: n.a.	1 L/d of SD Composition per 100 mL: 1.4 g carbohydrate 1.1 g fat 1.8 g protein 60 mg Ca 8 mg aglycone equivalents of genistein 6.3 mg aglycone equivalents of daidzein	1 L/d of CM Composition per 100 mL: 2.5 g carbohydrate 0.2 g fat 1.6 g protein 60 mg Ca	SBP, DBP, MBP	↓ SBP, DBP and MBP with SD vs. CM ↓ SBP, DBP and MBP with SD vs. baseline Urinary genistein excretion was negatively correlated with the ↓ in DBP
[21]	4-week, double-blind crossover trial (washout period 4 weeks)	20 patients with hypercholesterolemia (16 F, 4 M) Age: 59.5 ± 8.3 years BMI: 24.2 ± 3.5 kg/m^2^	500 mL/d of SD Composition per 100 mL: 1.9 g carbohydrate 2.9 g fat 5 g protein 12 mg Ca 5 mg genistein 5.5 mg daidzein 4.8 mg glycitein	500 mL/d of CM Composition: n.a.	Lipid profile (TC, LDL-C) and BW	⟷ Between CM and SD
[22]	6-week, randomized, double-blind, crossover trial (no washout period)	60 outpatients with primary hypercholesterolemia (45 F, 15 M) Age: 56 ± 1 ^§^ years BMI: 24.9 ± 0.3 ^§^ kg/m^2^	1 L/d of SD Composition per 100 mL: 0.5 g carbohydrate 1.8 g fat 2.5 g protein 9.1 mg Ca 5.0 mg genistein 3.3 mg daidzein 0.5 mg glycitein	1 L/d of CM Composition per 100 mL: 4.3 g carbohydrate 0 g fat 2.8 g protein 89.3 mg Ca	Lipid profile (TC, HDL-C, LDL-C, triacylglycerols) TBARs and BMI	↓ LDL-C with SD vs. baseline and CM ↑ HDL-C with SD vs. baseline and CM ↓ TBARs with SD vs. CM ⟷ Triacylglycerols, TC with SD vs. baseline, CM No significant BMI changes in CM and SD
[23]	16-week, randomized, placebo-controlled, parallel, double-blind trial	52 postmenopausal women SD group (*n* = 18) Age: 56.1 ± 4.4 ^§^ years BMI: 27.4 ± 6.2 ^§^ kg/m^2^ SD + Supplement group (*n* = 15) Age: 55.9 ± 3.5 ^§^ years BMI: 28.8 ± 5.4 ^§^ kg/m^2^ CTRL group (*n* = 19) Age: 55.4 ± 3.9 ^§^ years BMI: 27.5 ± 4.9 ^§^ kg/m^2^	706 mL/d of SD plus a placebo supplement Composition per 100 mL: 4.2 g carbohydrate 1.4 g fat 2.5 g protein 127.5 mg Ca 4.4 mg daidzein 5.3 mg genistein 0.5 mg glycitein 10.1 mg total isoflavones	(1) 706 mL/d of CM plus a placebo supplement (CTRL) Composition per 100 mL: 5.1 g carbohydrate 1.3 g fat 3.4 protein 127.5 mg Ca (2) 706 mL/d of CM plus 70 mg isoflavones (30 mg daidzein, 33 mg genistein, and 7 mg glycetin) in a supplement for 16 weeks Composition per 100 mL of CM: 5.1 g carbohydrate 1.3 g fat 3.4 g protein 127.5 mg Ca	Lymphocyte subsets (total T cells, T cytotoxic cells, T helper cells, ratio of Th to Tc cells, B cells, natural killer cells), cytokine production (IFN-γ, TNF-a, IL-2), CRP, lipid peroxidation (8-Isoprostane), DNA damage (8-OHdG) and BMI	A significant effect of group on percentage of B cells ↑ B cell populations in SD and supplement groups vs. CTRL Significant effects of group, week and group x week interaction on plasma 8-OHdg ↓ Plasma concentrations of 8-OHdg in SD and supplement groups vs. CTRL ⟷ For other parameters between CM and SD
[24]	16-week, randomized, parallel, double-blind, controlled trial	79 postmenopausal women SD group (*n* = 25) Age: 56.1 ± 0.9 ^§^ years BMI: 26.8 ± 1.2 ^§^ kg/m^2^ SD + Supplement group (*n* = 27) Age: 55.7 ± 0.7 ^§^ years BMI: 28.2 ± 0.9 ^§^ kg/m^2^ CTRL group (*n* = 27) Age: 56.4 ± 0.8 ^§^ years BMI: 28.5 ± 1.3 ^§^ kg/m^2^	706 mL/d of SD plus a placebo supplement Composition per 100 mL: 4.2 g carbohydrate 1.4 g fat 2.5 g protein 127.5 mg Ca 4.4 mg daidzein 5.3 mg genistein 0.5 mg glycitein 10.1 mg total isoflavones	(1) 706 mL/d of CM plus a placebo supplement (CTRL) Composition per 100 mL: 5.1 g carbohydrate 0.8 g fat 3.4 g/d protein 127.5 mg Ca (2) 706 mL/d of CM plus 70 mg isoflavones (30 mg daidzein, 33 mg genistein, and 7 mg glycetin) in a supplement for 16 weeks (supplement group) Composition per 100 mL of SD: 5.1 g carbohydrate 1.3 g fat 3.4 g protein 127.5 mg Ca	Selective attention (Stroop task), visual long-term memory (pattern recognition), short-term visuospatial memory (Benton Visual Retention Test), visuospatial working memory (Color Match Task), verbal working memory (Digit Ordering Task), verbal memory span (Forward Digit Span), spatial memory span (Corsi Block-Tapping)	↓ Verbal working memory (Digit Ordering Task) in SD group relative to baseline vs. soy supplement and CTRL groups ↑ Improvements in Color Match performance and in pattern recognition relative to baseline for all the groups ⟷ For other parameters between SD and CM
[25]	4-week, randomized, crossover trial (washout period ≥ 4 week)	28 hypercholesterolemic adults (22 F, 6 M) Age: 52 ± 9 years BMI: 26 ± 4 kg/m^2^	(1) 32 (946.3 mL) oz/d of WB Composition per 100 mL: 4.2 g carbohydrate 1.4 g fat 2.6 protein 13.2 mg aglycone equivalents of total isoflavones (6.9 genistein, 6.0 daidzein, 0.3 glycitein) (2) 28 (828.1 mL) oz/d of SPI made with soy protein isolate Composition per 100 mL: 4.7 g carbohydrate 1.1 g fat 3 g protein 4.7 mg aglycone equivalents of total isoflavones (3.0 genistein, 1.6 daidzein, 0.1 glycitein)	18.5 (547.1 mL) oz/d of organic CM Composition per 100 mL: 6.4 g carbohydrate 1.1 g fat 4.6 g protein	Lipid profile (triacylglycerols, HDL-C, LDL-C), insulin, and GLU	↓ LDL-C concentration with WB and SPI vs. CM ↑ LDL-C/HDL-C differences among WB and SPI groups vs. CM group ⟷ For other parameters between CM and SD
[26]	8-week, randomized, prospective, parallel, controlled trial	14 women with overweight/obesity SD group (*n* = 7) Age: 33.7 ± 6.3 years BMI: 38.4 ± 10.0 kg/m^2^ Skimmed CM group (*n* = 7) Age: 29.4 ± 11.0 years BMI: 33.9 ± 10.6 kg/m^2^	720 mL/d of SD Composition: n.a.	720 mL/d skimmed CM Composition: n.a.	BW, BF%, AC, BMI and FFM,	↓ Attenuating loss of FFM with CM, SD vs. baseline ↓ BMI, BW, BF% and AC with CM, SD vs. baseline ⟷ For other parameters between CM vs. SD
[27]	16-week, randomized, placebo-controlled, parallel, double-blinded trial	77 healthy postmenopausal women CTRL group (*n* = 27) Age: 55.7 ± 0.8 years BMI: 29.4 ± 1.3 kg/m^2^ SD group (*n* = 26) Age: 55.8 ± 0.9 years BMI: 27.5 ± 1.2 kg/m^2^ Supplement group (*n* = 24) Age: 54.8 ± 0.7 years BMI: 28.6 ± 1.2 kg/m^2^	706 mL/d of SD with placebo tablets (maltodextrin) Composition per 100 mL: 4.2 g carbohydrate 1.4 g fat 2.5 g protein 127.5 mg Ca 4.4 mg daidzein 5.3 mg genistein 0.5 mg glycitein 10.1 mg total isoflavones	(1) 706 mL/d of CM + placebo tablet (maltodextrin) Composition per 100 mL: 5.1 g carbohydrate 0.8 g fat 3.4 g/d protein 127.5 mg Ca (2) 706 mL/d of CM with isoflavone supplement (70 mg/d isoflavone; supplement composition: 15 mg daidzein, 17 mg genistein, and 3.5 mg glycitein) Composition per 100 mL: 5.1 g carbohydrate 0.8 g fat 3.4 protein 127.5 mg Ca	Serum TSH	⟷ For TSH between CM and SD
[28]	4-week, single-blind, randomized, parallel, controlled trial	31 postmenopausal women Vanilla SD group (*n* = 16) Age: 53.9 ± 3.7 years BMI: 25.4 ± 4.1 kg/m^2^ Reduced-fat CM group (*n* = 15) Age: 55.0 ± 3.1 years BMI: 26.3 ± 4.0 kg/m^2^	732 mL/d (3 servings of 244 mL) of SD Composition per 100 mL: 7.8 g carbohydrate 1.6 g fat 2.5 g protein	709.8 mL/d (3 servings of 236.6 mL) of CM Composition per 100 mL: 5.1 g carbohydrate 1.9 g fat 3.4 g protein	BW, markers of inflammation (TNF-α, IL-6, IL-1β) and oxidative stress (SOD, GPx, COX-2)	⟷ For the investigated parameters between CM vs. SD
[29]	4-week, two randomized, crossover, controlled, trials (washout period 4 weeks)	STUDY 1 33 (18 F, 15 M) normal to hypercholesterolemic subjects Age: 43.0 ± 2.4 years BMI: 29.1 ± 1.0 kg/m^2^	STUDY 1 720 mL/d low-fat PS-enriched SD Composition per 100 mL: 4.2 g carbohydrate 0.8 g fat 2.5 g protein	STUDY 1 720 mL/d CM Composition per 100 mL: 5.0 g carbohydrate 1.0 g fat 3.3 g protein	STUDY 1 TC, HDL-C LDL-C, TG	STUDY 1 ↓ TC and LDL-C with low-fat PS-enriched SD vs. CM ↓ LDL/HDL and TC/HDL ratios with low-fat PS-enriched SD vs. CM ⟷ HDL-C and TG between low-fat PS-enriched SD vs. CM In a subgroup of subjects with initial LDL-C > 3.4 mmol/L, ↓ TC concentrations and LDL-C with low-fat PS-enriched SD vs. CM
		STUDY 2 23 (13 F, 10 M) hypercholesterolemic subjects Age: 43.9 ± 0.3 years BMI: 30.0 ± 1.5 kg/m^2^	STUDY 2 720 mL/d moderate-fat PS-enriched SD Composition per 100 mL: 4.2 g carbohydrate 1.5 g fat 2.5 g protein	STUDY 2 720 mL/d CM Composition per 100 mL: 5.0 g carbohydrate 1.0 g fat 3.3 g protein	STUDY 2 TC, HDL-C, LDL-C, TG, cholesterol absorption and synthesis	STUDY 2 ↓ TC and LDL-C with moderate-fat PS-enriched SD vs. CM ↓ Fasting TG with moderate-fat PS-enriched SD vs. CM ↓ LDL/HDL and TC/HDL ratios with moderate-fat PS-enriched SD vs. CM ↓ Cholesterol absorption with moderate-fat PS-enriched SD vs. CM ⟷ Cholesterol synthesis and HDL-C between moderate-fat PS-enriched SD vs. CM
[30]	4-week, single-blind, randomized, parallel, controlled trial	31 postmenopausal women SD group (*n* = 16) Age: 53.9 ± 3.7 years BMI: 25.4 ± 4.1 kg/m^2^ CM group (*n* = 15) Age: 55.0 ± 3.1 years BMI: 26.3 ± 4.0 kg/m^2^	3 servings/d of vanilla SD Composition per serving: 6 g protein ~30 mg isoflavones	3 servings/d of reduced-fat CM Composition per serving: 8 g protein	Markers of inflammation (TNF-α, IL-1β, IL-6) and oxidative stress (SOD, GPx COX-2),	A group-by-time effect in the CM group: ↑ TNF-α post-supplementation, ↓ TNF-α in the postexercise period Time effects for plasma SOD and IL-6: ↓ SOD activity from pre-exercise to the 4 h time period ↑ IL-6 from pre-exercise to the 4 h time period No significant group, group-by-time interaction or time effects on other parameters
[31]	4-week, single-blind, randomized, parallel, controlled trial	32 postmenopausal women Vanilla SD group (*n* = 16) Age: 53.9 ± 3.7 years BMI: 25.4 ± 4.1 kg/m^2^ Reduced-fat CM (*n* = 16) Age: 54.9 ± 3.1 years BMI: 26.3 ± 3.8 kg/m^2^	732 mL/d (3 servings of 244 mL) of SD Composition per 100 mL: 7.8 g carbohydrate 1.6 g fat 2.5 g protein	709.8 mL/d (3 servings of 236.6 mL) of reduced-fat CM Composition per 100 mL: 5.1 g carbohydrate 1.9 g fat 3.4 g protein	Lipid profile (TC, TG, LDL, HDL) and BW	⟷ For the lipid profile variables between CM and SD No significant BW changes in CM and SD
[32]	6-week, randomized, open-label, crossover trial (washout period of 3 weeks)	23 overweight and obese women Age: 22.1 ± 2.7 years BMI: 28.1 ± 0.5 kg/m^2^	240 mL/d of SD Composition per 100 mL: 3.5 g carbohydrate 1 g fat 2 g protein 40 mg Ca	240 mL/d of CM Composition: n.a.	BW, WC, HC, … BMI, DBP and SBP	↓ SBP, DBP with SD vs. CM ⟷ For other parameters between CM vs. SD
[33]	8-week, randomized, parallel, controlled trial	85 healthy overweight or obese premenopausal women CTRL group (*n* = 20) Age: 38.3 ± 9.5 years BMI: 30.8 ± 3.1 kg/m^2^ Ca group (*n* = 22) Age: 35.8 ± 8.7 years BMI: 31.5 ± 4.1 kg/m^2^ CM group (*n* = 22) Age: 38.3 ± 10.4 years BMI: 30.0 ± 3.6 kg/m^2^ SD group (*n* = 21) Age: 37.5 ± 9.3 years BMI: 31.1 ± 4.1 kg/m^2^	660 mL/d (3 servings of 220 mL) of Ca-fortified SD Composition: n.a.	660 mL/d (3 servings of 220 mL) of low-fat milk CM Composition: n.a.	BW, WC, BMI, WHR, BFM and BF%	↓ BW, BMI, WC, WHR, BFM and BF% with CM and SD vs. baseline ↑ BW change (% of initial) in the CM vs. SD group ⟷ For other parameters between CM vs. SD
[34]	3-month, randomized, parallel, double-blind trial	64 overweight and obese male volunteers Age: 32 ± 7 years BMI: 29 ± 4 kg/m^2^	(1) 500 mL/d of LGS (49.5% β-conglycinin/6% glycinin) Composition per 100 mL: 4.6 g carbohydrate 1.4 g fat 2.8 g protein 139.8 mg Ca (2) 500 mL/d of conventional SD (26.5% β-conglycinin/38.7% glycinin) for 3 months Composition per 100 mL: 4.6 g carbohydrate 1.4 g fat 2.8 g protein 132 mg Ca The composition of isoflavones was comparable between SD and LGCS	500 mL/d of CM Composition per 100 mL: 3.4 g carbohydrate 1.4 g fat 2.8 g protein 89.2 mg Ca	Total bacterial abundance, *Bacteroides-Prevotella, Bifidobacterium* and *Lactobacillus,* bacterial diversity, richness and phylogenic abundance profile	↑ Total bacterial copy numbers with all treatments ↑ Copy number od *Bacteroides-Prevotella* with LGS vs. baseline ↓ Relative abundance of Firmicutes, copy number of *Bifidobacterium* and Firmicutes-to-Bacteroidetes ratio with LGCS and SD vs. baseline ↑ Copy number of *Lactobacillus* with CM vs. baseline ↓ Bacterial diversity and richness and ↑ relative abundance of members in the phylum Proteobacteria with LGS, SD and CM vs. baseline ↑ Relative abundance of Bacteroidetes with LGS and SD vs. baseline ⟷ For other parameters between CM vs. SD
[35]	18-month, randomized, parallel, open-label, controlled trial	98 postmenopausal women without osteoporosis CM group (*n* = 50) Age: 55.8 ± 4.1 years BMI: 24.9 ± 3.2 kg/m^2^ SD group (*n* = 50) Age: 56.1 ± 4.2 years BMI: 24.0 ± 3.0 kg/m^2^ CTRL group (*n* = 41) Age: 57.3 ± 4.3 years BMI: 24.5 ± 2.6 kg/m^2^	250 mL/d of Ca-fortified SD Composition per 100 mL: 1 g fat 2.6 g protein 1 g lactose 100 mg Ca 1.5–1.8 mg soy isoflavones	250 mL/d of CM Composition per 100 mL: 2.6 g casein 4.8–5 g lactose 4 g fat 100 mg Ca	BMD	↑ Hip and femoral neck BMD at 18 months with CM vs. baseline ⟷ Lumbar BMD among the three groups
[36]	4-week, randomized, non-blinded, crossover trial (washout period 2 weeks)	24 overweight and obese women Age: 37.7 ± 1.3 ^§^ years BMI: 31.1 ± 0.8 kg/m^2^	240 mL/d of SD Composition per 100 mL: 3.5 g carbohydrate 1 g fat 2.5 g protein 40 mg Ca	240 mL/d of CM Composition per 100 mL: 4.9 g carbohydrate 1.5 g fat 3.3 g protein 100 mg Ca	BW, BMI, WC, WHR, DBP, SBP, liver enzymes (AST, ALT), fibrinogen, FBS and insulin	↓ WC with SD vs. CM ⟷ For other parameters between CM vs. SD
[37]	4-week, randomized, crossover trial (washout period 2 weeks)	25 type 2 diabetic patients with nephropathy (15 F, 10 M) Age: 51 ± 10 years BMI: 28 ± 4 kg/m^2^	240 mL/d of SD Composition per 100 mL: 3.5 g carbohydrate 1 g fat 2.5 g protein 40 mg Ca	240 mL/d of CM Composition per 100 mL: 4.9 g carbohydrate 1.5 g fat 3.3 g protein 100 mg Ca	BW, markers of inflammation (TNF-α, IL-6, hs-CRP) and oxidative stress (MDA), fibrinogen and D-dimer	↑ Percent changes (reduction) in D-dimer levels with SD vs. CM ⟷ For other parameters between CM vs. SD
[38]	4-week, randomized, non-blinded, crossover trial (washout period 2 weeks)	24 non-menopausal overweight and obese women Age: 37.7 ± 1.3 years BMI: 30.9 ± 0.8 kg/m^2^	240 mL/d of SD Composition per 100 mL: 3.5 g carbohydrate 1 g fat 2.5 g protein 40 mg Ca	240 mL/d of CM Composition per 100 mL: 4.9 g carbohydrate 1.5 g fat 3.3 g protein 100 mg Ca	BW, markers of inflammation (Hs-CRP, IL-6) and lipid profiles (TG, TC, HDL-C, LDL-C)	↓ Serum LDL with SD vs. CM ⟷ For other parameters between CM and SD
[39]	4-week, single-blinded, randomized, parallel, controlled trial	31 postmenopausal women SD Group (*n* = 16) Age: 54 ± 4 years BMI: 25.4 ± 4.1 kg/m^2^ CM group (*n* = 15) Age: 55 ± 3 years BMI: 26.3 ± 4.0 kg/m^2^	732 mL/d (3 servings of 244 mL) of SD Composition per 100 mL: 7.8 g carbohydrate 1.6 g fat 2.5 g protein	720 mL/d (3 servings of 240 mL) of reduced-fat CM Composition per 100 mL: 5.0 g carbohydrate 1.9 g fat 3.3 g protein	Skeletal muscle mRNA expression of inflammatory (TNF-a, IL-1β, IL-6) and proteolytic (calpain 1, calpain 2, ubiquitin, E2, atrogin-1, muRF-1) markers	No group-by-time interactions for the parameters but significant main effects for both groups
[40]	4-week, randomized, non-blinded, crossover, controlled trial (washout period 2 weeks)	25 type 2 diabetic patients with nephropathy (15 F, 10 M)Age: 51 ± 10 years BMI: 28 ± 4 kg/m^2^	240 mL/d of SD Composition per 100 mL: 3.5 g carbohydrate 1 g fat 2.5 g protein 40 mg Ca	240 mL/d of CM Composition per 100 mL: 4.9 g carbohydrate 1.5 g fat 3.3 g protein 100 mg Ca	BW, DBP, SBP, markers of kidney function (blood urea nitrogen, proteinuria, urine/serum creatinine, GFR), lipid profiles (serum TC, TG, HDL-C, LDL-C), serum insulin, GLU and HbA1C	↓ SBP with SD vs. CM ⟷ For other parameters between CM and SD
[41]	8-month, randomized, parallel, controlled trial	57 healthy postmenopausal women SD group (*n* = 34) Age: 52.1 ± 3.1 years BMI: 28.7 ± 3.8 kg/m^2^ CM group (*n* = 23) Age: 51.4 ± 2.9 years BMI: 28.6 ± 4.1 kg/m^2^	500 mL/d SD Composition per 100 mL: 37.1 mg of isoflavones 28.9 mg genistein 8.3 mg daidzein	500 mL/d low-fat CM Composition: n.a.	Quality of life (domains: vasomotor, psychosocial, physical, and sexual)	↓ Mean score of vasomotor domain (improvement in quality of life) with SD, CM vs. baseline ↑ Mean score of sexual domain (deterioration in quality of life) with CM vs. baseline, SD ⟷ For other parameters between CM vs. SD
[46]	2-week, randomized double-blind, crossover trial (washout period 1 week)	8 participants (5 F, 3 M) Mean age: 43 (29–60) years BMI: n.a.	200 mL/d of SD Composition per 100 mL: 3.1 g carbohydrate 3.0 g fat 3.0 g protein 120 mg Ca	200 mL/d of CM Composition per 100 mL: 4.8 g carbohydrate 3.4 g fat 3.3 g protein 117 mg Ca	Enamel lesion depth (LDb, Lda LDb-Lda)	↓ LDb-Lda with SD vs. CM ↑ ΔZb–Δza with CM (remineralization of the pre-existing subsurface enamel lesions) vs. SD
**Reference**	**Study Design**	**Study Population ^a^**	**Almond Drink Intervention ^b^**	**Cow’s Milk Intervention ^b^**	**Health Outcome ^c^**	**Health-Related Findings ^c^**
[43]	Postprandial, randomized, non-blinded, crossover, controlled trial (washout period ≥ 1 week)	26 healthy adults (13 F, 13 M) Age: 23.0 ± 2.6 years BMI: 22.3 ± 1.5 kg/m^2^	250 mL of AD Composition per 100 mL: 3.6 g carbohydrate 1.2 g fat 0.4 g protein	250 mL of CM Composition per 100 mL: 4.8 g carbohydrate 1.2 g fat 3.6 g protein	Subjective appetite, insulin and PPG	⟷ For appetite between CM and SD Glucose/insulin iAUC ratio with AD vs. CM

Health-related findings report only significant results. Data are presented as means ± standard deviation. ^a^ n.a.: Not available; CTRL: control; W: women; M: men. ^b^ AD: almond drink; Ca: calcium; Ca-D: calcium-supplemented diet; CI-CM: co-ingestion of cow’s milk; CI-SD: co-ingestion of soy drink; CM: cow’s milk; LGS: low-glycinin soy drink; MD: milk diet; Na: sodium; Pi: phosphorus; P-CM: preload cow’s milk; P-SD: preload soy drink; PS: plant sterol; RD: rice drink; SD: soy drink; SPI: soy drink made using soy protein isolate; TLC: Adult Treatment Panel of the Third Report of the National Cholesterol Education Program Therapeutic Lifestyle Changes; WB: soy drink made using whole soybeans. ^c^ AC: abdominal circumference; ADMA: asymmetric dimethylarginine; ALT: alanine aminotransferase; ARG: arginine; AST: aspartate aminotransferase; AUC: area under the curve; AUCSR: shear rate AUC; BFM: body fat mass; BF%: body fat percentage; BMI: body mass index; BW: body weight; COX-2: cyclooxygenase-2; CRP: C-reactive protein; CVC: cutaneous vascular conductance; CYS: cysteine; CYSS: cystine; DBP: diastolic blood pressure; FBS: fasting blood sugar; FFM: fat-free mass; FI: food intake; FMD: brachial-artery-flow-mediated dilation; GFR: glomerular filtration rate; GI: glycemic indexes; GIP: glucose-dependent insulinotropic polypeptide; GLP-1: glucagon-like peptide-1; GLU: glucose; GPx: glutathione peroxidase; GSH: reduced glutathione; HC: hip circumference; HDL: high-density lipoprotein; Hs-CRP: high-sensitivity C-reactive protein; II: insulinemic index; IL-1β: interleukin-1β; IL-2: interleukin-2; IL-6: interleukin-6; IL-LDL: low-density lipoprotein; iAUC: incremental AUC; II: insulinemic index; LDa: lesion depth after exposure to the test products; LDb: initial lesion depth; LDL: low-density lipoprotein; MBP: mean blood pressure; MDA: malondialdehyde; muRF-1: muscle ring finger-1; NO: nitric oxide; PPG: postprandial glycemia; PTH: intact parathyroid hormone; RBF: red blood cell flux; SBP: systolic blood pressure; SOD: superoxide dismutase; S-Ca: serum calcium; S-Pi: serum phosphorus; TBARs: thiobarbituric-reactive substances; TC: total cholesterol; TG: triglycerides; TNF-α: tumor necrosis factor; TSH: thyroid-stimulating hormone; WC: waist circumference; WHR: waist-to-hip ratio; ΔZa: mineral loss after exposure to the test product; ΔZb: mineral loss before exposure to the test products; 8-OHdg: 8-hydroxy-2-deoxy-guanosine; ↑: significant increase; ⟷: not significant changes; ↓: significant decrease. ^§^ Mean ± SEM.

## Data Availability

The data presented in this study are available on request from the corresponding author.
